# Supplementary material for: Identification of Freezing-Responsive microRNAs and Their Targets in Chinese Jujube by Small RNA and Degradome Sequencing
Source: Int J Mol Sci. 2026 Sep 4;27(17):7912. doi: 10.3390/ijms27177912 (PMC13566890; doi:10.3390/ijms27177912)
Supplement: Supplementary file 1 [file ijms-27-07912-s001.zip › Supplementary Material S2/novel_miR_145.pdf]

Provisional ID	:	novel_miR_145
Score total	:	0.4
Score for star read(s)	:	-1.3
Score for read counts	:	0
Score for mfe	:	0.1
Score for randfold	:	1.6
Score for cons. seed	:	
Total read count	:	1919
Mature read count	:	1893
Loop read count	:	26
Star read count	:	0

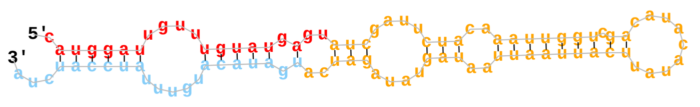

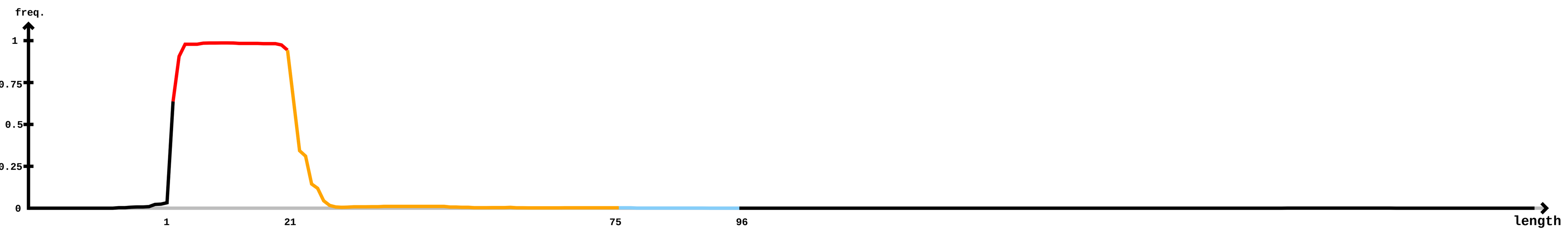

Mature **Star**

```
5'- aauccaaaucuuguuccuggcacauggauuguuuguaugaguaucgauucuacaaauuggucgacauacauauucauuaauuaauaguauagaucaugauacauguuuauccaucuaaacggcuagagagauaucccuucuauuuaagaaauagagaauauuuauagaugaguauuaauagaugaguaaagcguauccaaagaaacaaauagauuuuguuuccucguuuuuuuauuuauuuguucauacu -3'   exp
    .............((((((..((((((....(((((.(..(((....(((..(((((((.((........)))))))))..)))....)))..).)))))....))))))......))))).))((((...((((((((...)))))))).)))))........(((((.((((((((((((((((........((((((((.....))))))))..)))...)))))))))))))).)))))   reads   mm   sample
    .............uuccuggcacauggauugu...   1    0   LT-3
    .............uuccuggcacauggauuguC...   3    1   JCK-2
    .............uuccuggcacauggauuguC...   2    1   JCK-3
    ...............ccuggcacauggauuguuugua...   3    0   LCK-3
    ...............ccuggcacauggauuguuuguaugagu...   3    0   LCK-3
    ................cuggcacauggauuguuuguaugaguC...   3    1   LCK-3
    ..................ggcacauggauuguuuguaugagA...   2    1   LT-3
    ..................ggcacauggauuguuuguaugaguau...   2    0   LT-2
    ...................gcacauggauuguuuguaugagu...   1    0   LT-3
    ...................gcacauggauuguuuguaugagu...   1    0   LT-1
    ...................gcacauggauuguuuguaugagua...   20   0   LCK-3
    ...................gcacauggauuguuuguaugagua...   1    0   LT-1
    ...................gcacauggauuguuuguaugaguC...   1    1   LT-2
    ...................Acacauggauuguuuguaugaguau...   2    1   JCK-2
    ...................gcacauggauuguuuguaugaguaucgaC...   1    1   LT-2
    ....................cacauggauuguuuguaugaA...   1    1   LT-1
    ....................cacauggauuguuuguaugagua...   3    0   LT-1
    .....................acauggauuguuuguaugagu...   1    0   JCK-2
    .....................acauggauuguuuguaugagu...   1    0   JCK-3
    .....................acauggauuguuuguaugagu...   4    0   LT-3
    .....................acauggauuguuuguaugagua...   1    0   JCK-2
    .....................acauggauuguuuguaugagua...   3    0   LT-2
    .....................acauggauuguuuguaugagua...   1    0   LT-1
    .....................acauggauuguuuguaugaguauc...   1    0   JCK-1
    .....................acauggauuguuuguaugaguauc...   3    0   LT-3
    .....................acauggauuguuuguaugaguauc...   1    0   JCK-2
    .....................acauggauuguuuguaugaguauc...   1    0   JCK-3
    ......................cauggauuguuuguCuga...   1    1   LT-2
    ......................cauggauuguuuguaugC...   1    1   LT-2
    ......................cauggauuguuuguauga...   1    0   JCK-3
    ......................cauggauuguuuguauga...   1    0   LT-1
    ......................cCuggauuguuuguauga...   1    1   LT-2
    ......................cauggauuguuuguauga...   11   0   LT-2
    ......................cauggauuguuuguaugag...   44   0   LCK-3
```

Mature **Star**

aauccaaaucuuguuccuggca**cauggauuguuuguaugagu**aucgauucuacaaauuggucgacauacauauucauuaauuaauaguauagauca**ugauacauguuuauccaucua**aacggcuagagagauaucccuucuauuuaagaaauagagaauauuuauagaugaguauuaauagaugaguaaagcguauccaaagaaacaaauagauuuuguuuccucguuuuuuuauuuauuuguucauacu

cauggauuguuuguaugag	4	0	LT-1
cCuggauuguuuguaugag	1	1	LCK-3
cauggauuguuuguCugag	1	1	LCK-3
cauggauuguuuguaugag	4	0	LT-2
cauggauuguuuguaugag	3	0	JCK-1
cauggauuguuuguaugag	1	0	JCK-2
cauggauuguuuguaugag	3	0	LT-3
cauggauuguuuguaugagA	1	1	JCK-1
cauggauuguuuguaugagu	11	0	JT-1
cauggauuguuuguaugagu	41	0	LT-1
cauggauuguuuguCugagu	1	1	LCK-3
cCuggauuguuuguaugagu	2	1	LCK-3
cauggauuguuuguaugagu	4	0	JT-3
cauggauuguuuguaugCgu	1	1	LT-1
cauggauuguuuguaugCgu	1	1	LCK-3
cauggauuguuuguCugagu	1	1	LCK-2
cauggauuguuuguaugagu	17	0	LCK-1
cauggauuguuuguaugagA	1	1	LT-1
cauggauuguuuguaugagu	79	0	LT-2
cauggauuguuuguaugagu	14	0	JCK-3
cCuggauuguuuguaugagu	1	1	LT-2
cauggauuguuuguaugagu	49	0	LT-3
cauggauuguuNguaugagu	1	1	JT-1
cauggauuguuuguaugagu	15	0	LCK-2
cauggauuguuuguaugagu	18	0	JCK-1
cauggauuguuuguaugagu	4	0	JT-2
cauggauuguuuguaugagu	480	0	LCK-3
cauggauuguuuguaugagu	18	0	JCK-2
cCuggauuguuuguaugagua	2	1	LCK-3
cauggauuguuuguaugagua	17	0	LT-1
cauggauuguuuguaugagua	158	0	LCK-3
cauggauuguuuguCugagua	1	1	LT-2
cauggauuguuuguaugagua	10	0	JCK-2
cauggauuguuuguaugagua	11	0	LT-2
cauggauuguuuguaugagua	7	0	JCK-1
cauggauuguuuguCugagua	1	1	LCK-3
cauggauuguuuguaugagua	4	0	JCK-3
cauggauuguuuguaugagua	9	0	JT-2
cauggauuguuuguaugagua	15	0	LT-3
cauggauuguuuguCugagua	1	1	LT-1
cauggauuguuuguCugagua	1	1	JT-3
cauggauuguuuguaugagua	8	0	JT-3
cauggauuguuuguaugaguC	3	1	LT-3
cauggauuguuuguaugaguG	1	1	LT-1
cauggauuguuuguaugaguG	8	1	LCK-3
cauggauuguuuguaugaguG	1	1	LT-3
cauggauuguuuguaugaguau	1	0	LT-3
cauggauuguuuguaugaguau	2	0	LCK-3
cauggauuguuuguaugaguaA	1	1	LT-1
cauggauuguuuguaugaguau	1	0	LT-2
cauggauuguuuguaugaguaA	3	1	LT-3
cauggauuguuuguaugaguauc	7	0	JT-1
cauggauuguuuguaugaguauG	1	1	JCK-1
cauggauuguuuguaugaguauc	3	0	JCK-2
cauggauuguuuguaugaguauA	4	1	LT-3
cauggauuguuuguaugaguauc	8	0	LT-2
cauggauuguuuguaugaguauG	1	1	LT-1
cauggauuguuuguaugaguauc	2	0	LT-3
cauggauuguuuguaugaguauA	13	1	JCK-1
cauggauuguuuguaugaguauG	1	1	JCK-3
cauggauuguuuguaugaguauA	4	1	LT-1
cauggauuguuuguaugaguauA	9	1	LCK-3
cauggauuguuuguaugaguauc	8	0	JCK-3
cauggauuguuuguaugaguauc	5	0	LT-1
cauggauuguuuguaugaguauc	13	0	LCK-3
cauggauuguuuguaugaguauA	1	1	LT-2
cauggauuguuuguaugaguauA	10	1	JCK-2
cauggauuguuuguaugaguauc	5	0	JCK-1
cauggauuguuuguaugaguauA	9	1	JCK-3
cauggauCguuuguaugaguauc	1	1	LT-3

```
                      Mature                                                                    Star
aauccaaaucuuguuccuggcacauggauuguuuguaugaguaucgauucuacaaauuggucgacauacauauucauuaauuaauaguauagaucaugauacauguuuauccaucuaaacggcuagagagauaucccuucuauuuaagaaauagagaauauuuauagaugaguauuaauagaugaguaaagcguauccaaagaaacaaauagauuuuguuuccucguuuuuuauuuauuuguucauacu
......................cauggauuguuuguaugaguaucg        1   0   LT-3
......................cauggauuguuuguaugaguaucg        3   0   LT-2
......................cauggauuguuuguaugaguaucg        3   0   LT-1
......................cauggauuguuuguaugaguauAga       10  1   LCK-3
......................cauggauuguuuguaugaguaucga       1   0   LT-3
......................cauggauuguuuguaugaguaucga       2   0   LT-1
......................cauggauuguuuguaugaguaucgau      1   0   LT-1
......................cauggauuguuuguaugaguaucgaA      1   1   LT-1
......................cauggauuguuuguaugaguaucgaA      10  1   JCK-1
......................cauggauuguuuguaugaguaucgauu     1   0   LT-1
......................cauggauuguuuguaugaguaucgauu     2   0   LT-2
......................cauggauuguuuguaugaguaucgauucu   1   0   LT-2
.......................auggauuguuuguaugag             2   0   LT-2
.......................auggauuguuuguaugagC            1   1   LT-3
.......................auggauuguuuguaugagA            2   1   LT-3
.......................auggauuguuuguaugagu            2   0   JCK-3
.......................auggauuguuuguaugagu            1   0   JCK-1
.......................auggauuguuuguaugagG            1   1   LT-1
.......................auggauuguuuguaugagu            1   0   JCK-2
.......................auggauuguuuguaugagu            12  0   LCK-3
.......................auggauuguuuguaugagu            8   0   LT-3
.......................auggauuguuuguaugagA            2   1   JCK-2
.......................auggauuguuuguaugagu            2   0   LT-1
.......................auggauuguuuguaugagu            19  0   LT-2
.......................auggauuguuuguaugagua           7   0   LT-2
.......................auggauuguuuguaugagua           6   0   JCK-3
.......................auggauuguuuguaugagua           12  0   LT-3
.......................auggauuguuuguaugagua           6   0   LCK-3
.......................auggauuguuuguaugagua           3   0   JCK-2
.......................auggauuguuuguaugagua           5   0   JT-2
.......................auggauuguuuguCugagua           1   1   JT-1
.......................auggauuguuuguaugagua           2   0   LT-1
.......................auggauuguuuguaugaguC           1   1   LT-2
.......................auggauuguuuguaugagua           25  0   JT-3
.......................auggauuguuuguaugagua           2   0   JCK-1
.......................auggauuguuuguaugaguC           1   1   LT-3
.......................auggauuguuuguaugagua           13  0   JT-1
.......................auggauuguuuguaugaguaA          2   1   JT-2
.......................auggauuguuuguaugaguaA          7   1   JT-3
.......................auggauuguuuguaugaguaA          1   1   JCK-1
.......................auggauuguuuguaugaguau          3   0   LT-3
.......................auggauuguuuguaugaguau          5   0   JT-3
.......................auggauuguuuguaugaguau          1   0   JCK-2
.......................auggauuguuuguaugaguau          7   0   LCK-3
.......................auggauuguuuguaugaguau          2   0   JT-2
.......................auggauuguuuguaugaguaA          1   1   JCK-3
.......................auggauuguuuguaugaguauc         8   0   JCK-1
.......................auggauuguuuguaugaguauG         2   1   JCK-2
.......................auggauuguuuguaugaguauc         7   0   LT-3
.......................auggauuguuuguaugaguauA         3   1   LT-3
.......................auggauuguuuguaugaguauc         28  0   LCK-3
.......................auggauuguuuguaugaguauA         11  1   JCK-3
.......................auggauuguuuguaugaguauA         18  1   JCK-1
.......................auggauuguuuguaugaguauA         8   1   JCK-2
.......................auggauuguuuguaugaguauA         6   1   JT-2
.......................auggauuguuuguaugaguauA         4   1   LT-1
.......................auggauuguuuguaugaguauc         8   0   LT-2
.......................Cuggauuguuuguaugaguauc         1   1   LT-3
.......................auggauuguuuguaCgaguauc         1   1   LT-3
.......................auggauuguuuguaugaguauc         22  0   JT-3
.......................auggauuguuuguaugaguauA         9   1   LCK-3
.......................auggauuguuuguaugaguauc         10  0   JCK-2
.......................auggauuguuuguaugaguauc         12  0   LT-1
.......................auggauuguuuguaugaguauc         21  0   JCK-3
.......................auggauuguuuguaugaguauc         19  0   JT-2
.......................auggauuguuuguaugaguauG         1   1   JCK-1
.......................auggauuguuuguaugaguauA         3   1   JT-3
.......................auggauuguuuguaugaguaucg        7   0   LT-2
.......................auggauuguuuguaugaguaucg        1   0   LT-3
.......................auggauuguuuguaugaguaucg        1   0   LT-1
```

```
                       Mature                                                             Star
aauccaaaucuuguuccuggcacauggauuguuuguaugaguaucgauucuacaaauuggucgacauacauauucauuaauuaaauaguauagaucaugauacauguuuauccaucuaaacggcuagagagauaucccuucuauuuaagaaauagagaauauuuauagaugaguauuaauagaugaguaaagcguauccaaagaaacaaauagauuuuguuuccucguuuuuuuauuuauuuguucauacu
.......................auggauuguuuguaugaguaucg        6    0    JCK-2
.......................auggauuguuuguaugaguaucg        6    0    LCK-3
.......................auggauuguuuguaugaguaucga       8    0    LT-3
.......................auggauuguuuguaugaguaucga       20   0    LCK-3
.......................auggCuuguuuguaugaguaucga       1    1    LT-2
.......................auggauuguuuguaugaguaucga       16   0    JCK-3
.......................auggauuguuuguaugaguaucga       15   0    JCK-2
.......................auggauuguuuguaugaguaucga       5    0    JT-3
.......................auggauuguuuguaugaguaucga       6    0    JT-2
.......................aNggauuguuuguaugaguaucga       1    1    LT-2
.......................auAgauuguuuguaugaguaucga       1    1    LT-2
.......................auggauuguuuguaugaguaucga       4    0    JCK-1
.......................auggauuguuuguaugaguaucgG       1    1    LT-2
.......................auggauuguuuguaugaguaucga       30   0    LT-2
.......................auggauuguuuguaugaguaucga       11   0    LT-1
.......................auggauuguuuguaugaguaucgaA      5    1    JCK-3
.......................auggauuguuuguaugaguaucgaA      8    1    LT-2
.......................auggauuguuuguaugaguaucgaA      3    1    LT-1
.......................auggauuguuuguaugaguaucgaC      1    1    JCK-1
.......................auggauuguuuguaugaguaucgau      2    0    LCK-3
.......................auggauuguuuguaugaguaucgaG      1    1    LT-1
.......................auggauuguuuguaugaguaucgaA      3    1    JCK-1
.......................auggauuguuuguaugaguaucgaG      6    1    LCK-3
.......................auggauuguuuguaugaguaucgaC      1    1    JCK-2
.......................auggauuguuuguaugaguaucgaA      1    1    LT-3
.......................auggauuguuuguaugaguaucgaA      4    1    JCK-2
.......................auggauuguuuguCugaguaucgau      1    1    LCK-3
.......................auggauuguuuguaugaguaucgauA     2    1    JCK-2
.......................auggauuguuuguaugaguaucgauu     1    0    JCK-3
.......................auggauuguuuguaugaguaucgauA     2    1    LT-2
.......................auggauuguuuguaugaguaucgauu     1    0    JCK-1
.......................auggauuguuuguaugaguaucgauuc    2    0    LT-1
.......................auggauuguuuguaugaguaucgauuc    1    0    LT-3
........................uggauuguuuguaugagu            3    0    LT-3
........................uggauuguuuguaugagu            3    0    LT-1
........................uggauuguuuguaugagA            1    1    LT-3
........................uggauuguuuguaugagua           6    0    LT-3
........................uggauuguuuguaugagua           9    0    LCK-3
........................uggauuguuuguaugagua           2    0    JT-2
........................uggauuguuuguaugagua           1    0    LT-2
........................uggauuguuuguaugagua           4    0    JCK-3
........................uggauuguuuguaugagua           2    0    JT-3
........................uggauuguuuguCugagua           1    1    JT-2
........................uggauuguuuguaugagua           3    0    JCK-2
........................uggCuuguuuguaugaguau          1    1    JT-1
........................uggauuguuuguaugaguau          1    0    LT-3
........................uggauuguuuguaugaguau          14   0    JT-1
........................uggauuguuuguaugaguau          4    0    LCK-3
........................uggauuguuuguCugaguau          1    1    JT-1
........................uggauuguuuguaugaguaA          1    1    JT-3
........................uggauuguuuguaugaguau          1    0    JCK-2
........................uggauuguuuguaugaguaA          3    1    JT-2
........................uggauuguuuguaugaguauc         9    0    LT-3
........................uggauuguuuguaugaguauc         1    0    JCK-3
........................uggauuguuuguaugaguauA         1    1    LT-1
........................uggauuguuuguaugaguauc         2    0    LT-2
........................uggauuguuuguaugaguauc         3    0    JCK-2
........................uggauuguuuguaugaguauA         3    1    JCK-2
........................uggauuguuuguaugaguauc         7    0    LT-1
........................uggauuguuuguaugaguauA         3    1    LT-2
........................uggauuguuuguaugaguaucg        2    0    LT-2
........................uggauuguuuguaugaguaucg        1    0    LT-1
........................uggauuguuuguaugaguaucg        2    0    JCK-1
........................uggauuguuuguaugaguaucg        2    0    JCK-3
........................uggauuguuuguaugaguaucg        2    0    LT-3
........................uggauuguuuguaugaguaucg        5    0    JT-3
........................uggauuguuuguaugCguaucg        1    1    LT-2
........................uggauuguuuguaugaguaucg        1    0    JCK-2
........................uggauuguuuguaugaguaucg        7    0    LCK-3
........................uggauuguuuguaugaguaucg        1    0    JT-2
```

```
                    Mature                                                          Star
aauccaaaucuuguuccuggcacauggauuguuuguaugaguaucgauucuacaaauuggucgacauacauauucauuaauuaauaguauagaucaugauacauguuuauccaucuaaacggcuagagagauaucccuucuauuuaagaaauagagaauauuuauagaugaguauuaauagaugaguaaagcguauccaaagaaacaaauagauuuuguuuccucguuuuuuuauuuauuuguucauacu
........................uggCuuguuuguaugaguaucg............................................................................................................................................................................................................	1	1	JCK-2
........................uggauuguuuguaugaguaucga...........................................................................................................................................................................................................	6	0	LT-1
........................uggauuguuuguaugaguaucga...........................................................................................................................................................................................................	1	0	JCK-1
........................uggauuguuuguaugaguaucga...........................................................................................................................................................................................................	5	0	LT-2
........................uggauuguuuguaugaguaucga...........................................................................................................................................................................................................	5	0	LT-3
........................uggauuguuuguaugaguaucgaA..........................................................................................................................................................................................................	3	1	LCK-3
........................uggauuguuuguaugaguaucgau..........................................................................................................................................................................................................	3	0	LT-1
........................uggauuguuuguaugaguaucgau..........................................................................................................................................................................................................	9	0	LCK-3
........................uggauuguuuguaugaguaucgauu.........................................................................................................................................................................................................	1	0	LT-3
........................uggauuguuuguaugaguaucgauu.........................................................................................................................................................................................................	1	0	LT-1
...........................auuguuuguaugaguaucga...........................................................................................................................................................................................................	4	0	LCK-3
...........................auuguuuguaugaguaucgauu.........................................................................................................................................................................................................	1	0	LT-2
...........................Uuuguuuguaugaguaucgauu.........................................................................................................................................................................................................	7	1	LCK-3
...........................auuguuuguaugaguaucgauucu.......................................................................................................................................................................................................	1	0	LT-3
............................uuguuuguaugaguaucgauu.........................................................................................................................................................................................................	1	0	JCK-1
............................uuguuuguaugaguaucgauuc........................................................................................................................................................................................................	1	0	JCK-3
..............................guuuguaugaguaucgau..........................................................................................................................................................................................................	1	0	JCK-3
................................................ucuacaaauuggucgacaua......................................................................................................................................................................................	8	0	LCK-3
.................................................cuacaaauuggucgacauacaua..................................................................................................................................................................................	1	0	LCK-3
...................................................acaaauuggucgacauacaua..................................................................................................................................................................................	4	0	LCK-3
....................................................caaauuggucgacauaca....................................................................................................................................................................................	3	0	LCK-3
....................................................caaauuggucgacauacauauucauuaau.........................................................................................................................................................................	1	0	JCK-2
.......................................................auuggucgacauacauauucauGa...........................................................................................................................................................................	1	1	JCK-1
.........................................................uggucgacauacauauucauGa...........................................................................................................................................................................	4	1	LT-2
...........................................................................auuaauuaauaguauagaucauga.......................................................................................................................................................	1	0	JCK-1
..............................................................................aauuaauaguauagaucauga.......................................................................................................................................................	1	0	JCK-1
..............................................................................aauuaauaguauagaucauga.......................................................................................................................................................	2	0	LT-2
.......................................................................................uauagaucaugauacauguuuauc...........................................................................................................................................	1	0	LT-2
...............................................................................................................................................................................................................aauagauuuuguuuccuc.........................	1	0	JCK-3
```
